# Perceptions and Use of Generative Artificial Intelligence in Medical Students: A Multicenter Survey

**DOI:** 10.1177/23821205251391969

**Published:** 2025-10-29

**Authors:** Cecilia Tran, Brett N. Hryciw, Sean William Moore, Alan Chaput, Andrew John Ervine Seely

**Affiliations:** 1 Department of Medicine, Faculty of Medicine, 12365 University of Ottawa, Ottawa, Ontario, Canada; 2 Department of Critical Care, 27337 The Ottawa Hospital, Ottawa, Ontario, Canada; 3 Emergency Medicine Section, Division of Clinical Sciences, 26627 Northern Ontario School of Medicine University, Thunder Bay, Ontario, Canada; 4 Department of Anesthesiology and Pain Medicine, 27337 The Ottawa Hospital, University of Ottawa, Ottawa, Ontario, Canada; 5 Division of Thoracic Surgery, Department of Surgery, 12365 The Ottawa Hospital, Ottawa, Ontario, Canada; 6 Divisions of Thoracic Surgery and Critical Care Medicine, University of Ottawa, Ottawa, Ontario, Canada

**Keywords:** artificial intelligence, medical education, undergraduate training, survey, large language models

## Abstract

**Introduction:**

Generative artificial intelligence (AI) has transformative potential in medical training, and its role in medicine holds drastic implications for patients, healthcare providers, and society; however, its current use by medical students is unknown. The study aims to characterize the use, frequency of use, and perceptions of generative AI by Canadian medical students.

**Methods:**

A cross-sectional survey was distributed to 6 medical schools in Ontario, Canada, to investigate how medical students use generative AI in education, clinical settings, and for communication, and to assess the perceived barriers and enablers that influence their use.

**Results:**

A total of 167 respondents completed the survey (60.8% female, 69.3% in first and second year), and over 78.9% of respondents reported using generative AI, with ChatGPT being the most popular model; 53.0% of respondents were frequent users and reported using generative AI tools at least once a week. In clinical settings, students report using generative AI for learning and reviewing medical content, summarizing clinical guidelines, and generating differential diagnoses; 92.8% of students were willing to learn how to use generative AI to integrate it into their future clinical practice. At the same time, most medical students appreciated the limitations of generative AI in terms of its risk for inaccuracy (91.6%) and bias (78.9%); 75.9% of participants agreed that generative AI should be implemented as a resource or formal teaching topic in medical training.

**Discussion:**

The findings of this study may help guide medical education institutions in adapting curricula and developing policies to promote the ethical and appropriate use of generative AI in medicine.

## Introduction

The introduction of generative artificial intelligence (AI) built on large language models (LLMs) is forging a paradigm shift in human craftsmanship and intelligence. Large language models have demonstrated a remarkable capacity to process unstructured human language commands and respond with novel, contextually relevant text outputs.^1^ OpenAI's ChatGPT has been a large driving force behind the notoriety and discourse surrounding LLMs, as this publicly accessible dialogue interface has an estimated 200 million active users.^2^ ChatGPT is one of many dialogue interfaces in this flourishing space alongside other models, including Google Gemini, Perplexity AI, Glass AI, and more. The potential and ubiquity of generative AI have profound implications for future physicians and healthcare.^3,4^ Numerous studies have explored the potential utility of generative AI in medical education, boasting its abilities to accelerate complex, time-intensive tasks such as summarizing medical literature, creating personalized multiple-choice questions, and constructing clinical vignettes.^5–7^ In clinical settings, generative AI can facilitate clinical decision-making through the generation of differential diagnoses, investigation algorithms, and management plans. Medical trainees can greatly benefit from generative AI to navigate the rigorous demands and abundance of information involved in medical training. However, the use of generative AI in medicine has raised concerns. The outputs can be misleading, inaccurate, or biased, posing risks to patient safety.^7^ The overreliance of generative AI by learners may lead to plagiarism and declining critical thinking skills.^8^ Owing to these concerns, the use of generative AI in medicine must be cautioned and should be implemented in a manner that helps rather than harms clinical decision-making and improves patient safety.

Despite numerous studies highlighting the potential of generative AI in medicine, the understanding of how medical students use generative AI in their education is limited. While previous research has reported the perceptions and beliefs of medical students toward generative AI,^9–12^ this does not provide sufficient guidance for medical institutions to develop policies and curricular changes to responsibly address the real-world use of generative AI. The aim of this study is to address this gap in the literature and survey medical students on their exact use, frequency, and perceptions of generative AI in medical training across different centers in Ontario, Canada. The findings of this study are intended to support further consideration into how medical schools may embrace generative AI.

## Methods

A systematic approach was used to develop the questionnaire, which aimed to investigate the use of generative AI, and attitudes and perceptions surrounding its application in medical education, communication, and clinical workflows. The survey items were generated through a literature review of studies exploring medical students’ perceptions and utilization of AI,^10–15^ as well as the broader applications of AI in medicine.^1,6,7,16^ The questionnaire was first pretested through a process of inclusion and exclusion based on binary and general feedback provided by 2 staff physicians (BNH, AJES). Based on this feedback, the questionnaire was revised to improve clarity, minimize confusion, and ensure alignment with the study objectives. It was then pilot tested with 5 medical students, 1 resident, and 2 staff physicians. Participants in the pilot testing agreed that the questionnaire was clear, easy to complete, and that the response time was appropriate.

This survey focuses on the text-to-text and text-to-image models of generative AI, as these models are the most prominent and frequently cited in the literature. The survey consists of 27 items divided into 4 sections. The “Introduction” section outlined the implied consent form. The “Methods” section explores the utilization of generative AI and details the model type, version (free or premium), frequency, and language it is used in. The participants are asked to indicate how they use generative AI in clinical and educational settings and for communication. “Statistical Analysis” section investigates the perceptions and attitudes medical students have toward generative AI. The questions in this section explore student interest in integrating generative AI into medical education curricula and how students anticipate integrating generative AI into their future practices. This section also examines the enabling factors and barriers to the use of this technology in medicine. The multiple-choice questions were single choice responses, unless explicitly stated to “select all,” which indicates multiple-choice responses were allowed. The “Results” section collects demographic information from participants, including age, gender, level of medical education, institution, and race. The “Discussion” section presents an open forum that allows students to provide their recommendations on how institutions should respond to the emergence of generative AI. The complete survey is available in Supplemental File 1: Survey. The open survey was administered digitally on Microsoft Forms.

The cross-sectional observational study was conducted at the Ottawa Hospital Research Institute, Canada. This study conforms to the Strengthening the Reporting of Observational Studies in Epidemiology guidelines^17^ (Supplemental File 2). The questionnaire was distributed to the 6 medical schools in Ontario operating at the time of the survey period: McMaster University, the Northern Ontario School of Medicine University, Queen's University, the University of Toronto, the University of Ottawa, and Western University. The inclusion criteria were any medical students enrolled in an Ontario medical school at the time of survey dissemination. There were no exclusion criteria. The Ottawa Hospital Research Institute approved this study on May 2, 2024. This study adhered to the Declaration of Helsinki. The survey was emailed for dissemination to the Vice Deans of undergraduate medical education at each medical school in Ontario. The 6 medical institutions operating during the study period participated in the study, with 3839 medical students available as eligible study participants. For 4 out of the 6 schools, 2 emails were sent to recruit medical students of all years to participate in the survey. The first email was sent when the survey period began, and the second reminder email was sent 2 weeks before the survey was closed. For the other 2 schools, the survey was embedded in an electronic newsletter or posted on an online research forum. The survey was open for a total of 4 weeks at each institution. Participation was voluntary and anonymous. Implied consent was obtained at the start of the survey. We collected survey data from May 2024 to October 2024.

###  Statistical Analysis

All survey data collected were included in the statistical analysis using Microsoft Excel (Redmond, United States). Descriptive statistics were performed on survey responses, including the total number and percentages. Free-text responses were analyzed according to Kiger and Varpio's 6 step framework for thematic analysis.^18^

## Results

**Figure 1.**
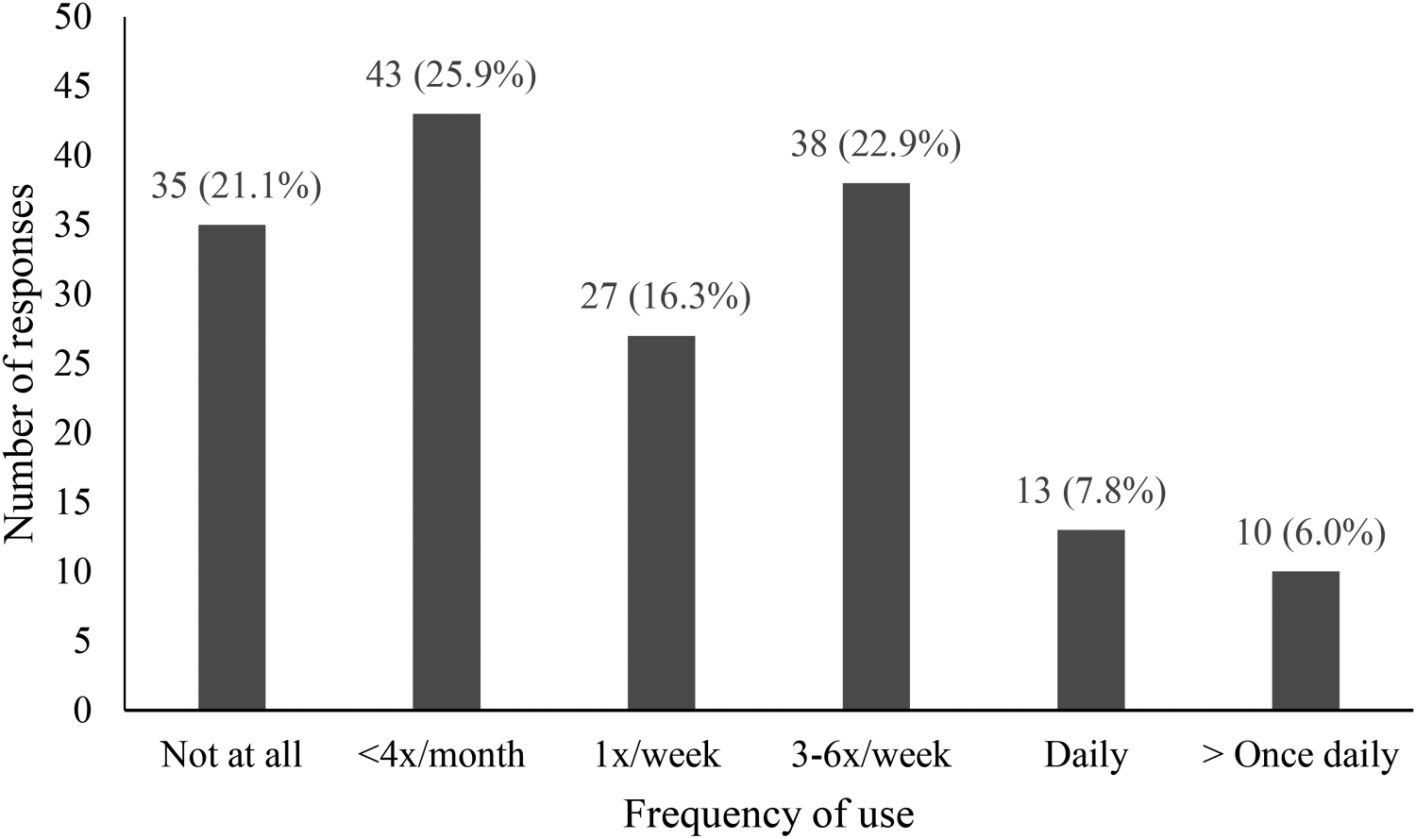
Frequency of Generative AI Use by Medical Students (n=166) for Both Medical School and Nonmedical School-Related Purposes.

A total of 167 medical students completed the survey (overall response rate = 4.3%), and the completeness rate was 99.4% (n = 166). Over half of the participants were women (n = 101, 60.8%), and 69.3% of the respondents were in preclerkship years (the first or second year of medical school). The participant demographic characteristics are outlined in Table 1. Most participants (n = 131, 78.9%) were active users of generative AI, whereas 21.1% (n = 35) were not. The free version of ChatGPT, which was ChatGPT-4o at the time of survey release (n = 119, 90.8%), was the most popular LLM used (Supplemental File 3). Figure 1 describes the frequency of generative AI use. Over half the respondents (53.0%) report using generative AI at least once a week for both medical school and nonmedical school-related purposes. English is overwhelmingly the main language in which generative AI tools are used in (n = 129, 95.4%), with a small subset using generative AI in French (n = 5, 3.8%) and in Spanish (n = 1, 0.8%); 11.4% of participants paid for a subscription to use the premium version(s) of generative AI. Of those who did not pay for a subscription, they cited the following reasons: do not use generative AI enough (n = 70, 63.6%), too expensive (n = 48, 43.6%), and little added value to the paid versions (n = 35, 31.8%).

**Table 1.** Demographic Characteristics of Respondents (n = 166).

Characteristics		Response n (%)
Age	18-24	101 (60.8%)
	25-34	62 (37.3%)
	35-45	1 (0.6%)
	No response	2 (1.2%)
Gender	Female	101 (60.8%)
	Male	60 (36.1%)
	Nonbinary	3 (1.8%)
	No response	3 (1.8%)
Level of education	Year 1	54 (32.5%)
	Year 2	61 (36.7%)
	Year 3	41 (24.7%)
	Year 4	8 (3.6%)
	No response	4 (2.4%)
Race	Arab	7 (4.2%)
	Black	5 (3.0%)
	Chinese	31 (18.6%)
	Filipino	4 (2.4%)
	Japanese	1 (0.6%)
	Korean	3 (1.8%)
	Latin American	2 (1.2%)
	South Asian (eg, East Indian, Pakistani, Sri Lankan)	26 (15.6%)
	Southeast Asian (eg, Vietnamese, Cambodian, Laotian, Thai)	5 (3.0%)
	West Asian (eg, Iranian, Afghan)	9 (5.4%)
	White	85 (50.9%)
	No response	8 (4.8%)
Institution	McMaster University	58 (34.9%)
	Northern Ontario School of Medicine University	17 (10.2%)
	Queen's University	1 (0.6%)
	University of Ottawa	38 (22.9%)
	University of Toronto	30 (18.1%)
	Western University	17 (10.2%)
	No response	5 (3.0%)

**Figure 2.**
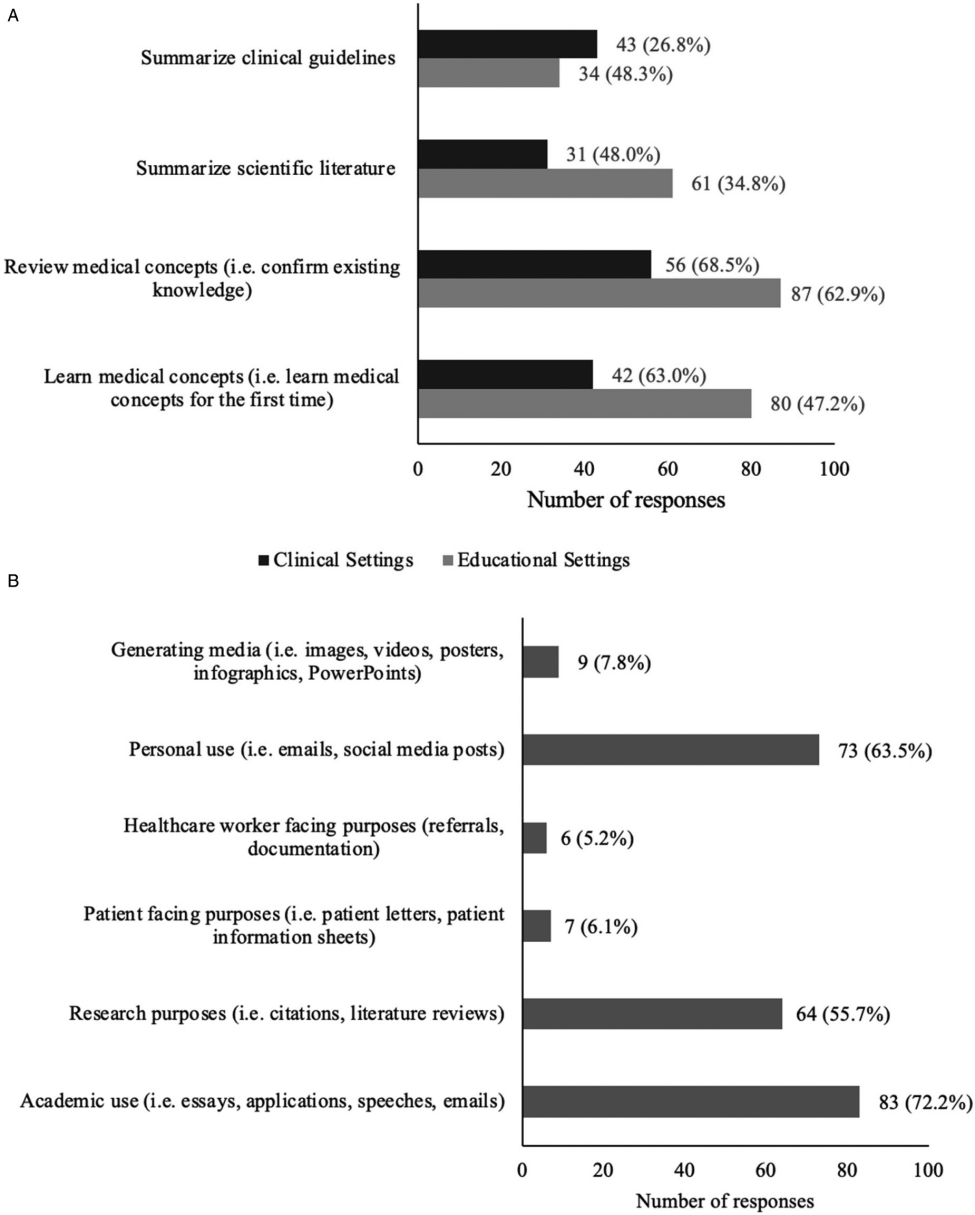
Medical Student Use of Generative AI: Applications in (A) Clinical (n=90) and Educational Settings (n=127), and for (B) Communication Purposes (n=115).

The most common use of generative AI in medical education was to review (n = 87, 68.5%) or learn (n = 80, 63.0%) medical content. Beyond knowledge consolidation, many students utilized generative AI as a study tool for examinations through the creation of study guides and summaries (n = 53, 41.7%), through self-testing as a question-bank generator (n = 44, 34.6%), and a diagram generator (n = 6, 4.7%). The use of generative AI as a learning tool in clinical settings was lower than that in educational settings, as shown in Figure 2. Other uses of generative AI in clinical contexts were to generate differential diagnoses (n = 41, 46.1%) and to support clinical decision-making (19, 21.3%). Figure 2 describes the applications of generative AI across 3 areas: education, clinical settings, and communication.

**Figure 3.**
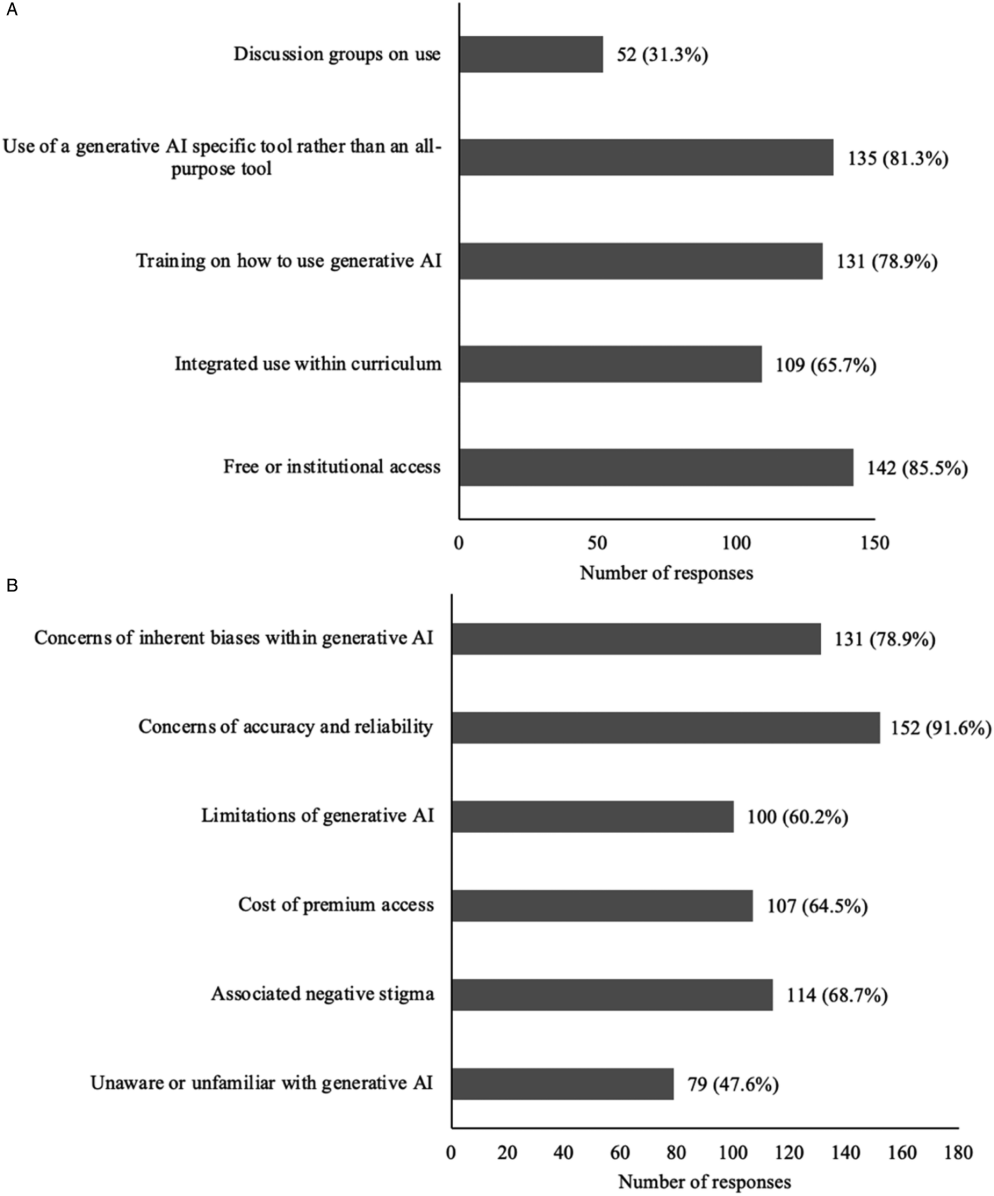
(A) Enablers Medical Students Perceived to Encourage Their Use of Generative AI in the Future (n=165), (B) Barriers Medical Students Perceived to Discourage Their Use of Generative AI in the Future (n=164).

Most respondents (n = 126, 75.9%) believed that AI should be implemented as a resource or be taught in medical school or residency. An even greater number of participants (n = 154, 92.8%) were willing to learn how to use generative AI and integrate it into their future practice. Table 2 describes the medical applications that the respondents anticipated using generative AI to assist with in the future. Despite the enthusiasm for generative AI, many medical students were still able to identify the limitations associated with its use. Many reported concerns over the accuracy and reliability (n = 152, 91.6%), concerns of the inherent biases (n = 131, 78.9%), and acknowledged the negative stigma associated with its use (n = 114, 68.7%). The enablers and barriers that participants perceived as influencing their use of generative AI in the future are described in Figure 3.

**Table 2.** Medical Student Perceptions Toward the Use of Generative AI in Medicine.

Questionnaire Item	Yes	No
Q13. Do you think generative AI should be implemented as a resource or be taught in the undergraduate medical education (UGME) or postgraduate medical education (PGME) curriculum? (n = 164)	126 (75.9%)	38 (22.9%)
Q14. Are you willing to learn how to use generative AI and integrate it into your practice in the future in any capacity? (n = 165)	154 (92.8%)	11 (6.6%)
*Written material for patient-facing purposes (ie, patient letters, patient information sheets)*	131 (85.1%)	
*Written material for health care provider-facing purposes (ie, referrals, encounter notes)*	138 (89.6%)	
*Generating differential diagnoses*	88 (57.1%)	
*Approach to patient assessment*	84 (54.5%)	
*Management and treatment*	74 (48.1%)	

In the free-text responses, the following themes were identified as topics medical students wanted formal education on (Table 3): validated and appropriate applications of generative AI in clinical settings (n = 29) and the use of generative AI as a study tool (ie, objective structured clinical exam preparation (OSCE), question bank generator, and a study guide) (n = 12). The following themes identified as areas of concern regarding the use of generative AI in medicine are described in Table 4. Many students expressed concern that the use of generative AI may negatively impact learning and critical thinking. Respondents shared recommendations for how they believe generative AI teaching can be delivered and implemented into the medical curriculum (Table 5).

**Table 3.** Medical Student Free-Text Recommendations on Desired Educational Topics for Generative AI in Medical Training (n = 54).

Themes	Examples	N
Ethical and medicolegal implications of generative AI	“Privacy/confidentiality in AI tools”	2 (3.7%)
Utility of generative AI as a study tool	“How to use AI… as a study resource during pre-clerkship”	4 (7.4%)
Validated and appropriate applications of generative AI in clinical settings	“I would also like to see updates of how AI is being used in medical practice, and have these tools included in clinical skills practice…”	14 (25.9%)
Utility of generative AI for documentation in clinical settings (ie, SOAP notes, d/c summaries)	“Provide more information (literally anything!) especially how to use programs for clinical notes and patient charting.”	7 (13.0%)
Foundational knowledge of LLMs and machine learning	“I’d like a foundations course on AI to learn the basics (ie, structure, functions, applications). It would be useful to have something akin to a “systemic review” of AI in medicine.”	4 (7.4%)
Best practices of LLM use (ie, prompt engineering, citation checking)	“How to interpret and use AI-based clinical decision support tools”	9 (16.7%)
Limitations and biases of LLMs	“I believe it's important for institutions to make students aware of the limitations of AI … it can also have inherent biases in its dataset, be based on non-peer reviewed sources, make incorrect statements that seem correct, and more.”	9 (16.7%)
Overview of domain-trained LLMs in medicine	“… updates on new medicine-specific models in future”	2 (3.7%)
Utility of generative AI in research	“Using AI to support literature searches/summarizing research”	3 (5.5%)

**Table 4.** Medical Student Free-Text Responses to Concerns Over the Use of Generative AI in Medical Training (n = 89).

Themes	Examples	N
Concerns of reliability, hallucinations, misinformation	“Potentially unreliable information, recommendations may not be based on peer-reviewed sources”	39 (43.8%)
Ethical implications and confidentiality concerns	“There is very little legislation governing AI use and guidelines for practice”	6 (6.7%)
Concerns for impaired learning, critical thinking, overreliance	“Lack of development of core medical skills, suppression of creativity”	29 (53.7%)
Environmental concerns, sustainability	“AI, particularly generative AI, can be very resource intensive (image generation) and massively consume electricity, the leading companies are not ethical in data sourcing”	3 (3.3%)
Concerns of bias	“the leading companies are not ethical in data sourcing… and in its current state it suffers from racial biases as a result of limited data”	8 (9.0%)
Accountability and safety of use in clinical settings	“I think that the use of AI in medicine suffers from the same problem as in academia. When something goes wrong (eg, plagiarized content, incorrect information, unjustified diagnosis), who is held accountable? And if we are replacing the hard tasks with AI (eg, generating plausible diagnoses), how will learners develop essential skills? There's also problem of garbage in, garbage out, where biased data can yield biased results. See, the example of a group that accidentally taught a machine learning program to associate tumors with rulers: https://doi.org/10.1016/j.jid.2018.06.175”	4 (4.5%)

**Table 5.** Medical Student Free-Text Responses of Recommendations for How to Implement AI in Undergraduate Medical Education (n = 43).

Themes	Examples	N
Discussion groups/open forums	“…Discussions and open forums would be appropriate for stimulating student engagement”	1 (2.3%)
Integrate into case-based learning	“Give us practice questions or scenarios based on things like patient interactions, coming up with differentials, etc and ask us to use AI to solve it”	3 (7.0%)
Integrate into clinical skills tutorials	“Using AI powered SOAP note tools within the clinical skill sessions with standardized patients”	3 (7.0%)
Workshops	“A lecture series or interactive workshops on the integration of AI into clinical medicine could be given.”	10 (23.3%)
Training on how to use it as a study tool (ie, question bank generator, Observed Structured Clinical Examination practice, image generation)	“Provide training to allow for more standardized use; OSCE prep; exam prep; image-based look-up tool”	6 (13.9%)
Training on appropriate/validated applications in clinical settings	“When learning topics where AI systems are currently being implemented into clinical practice, I think this should be included in our learning. For example, radiology is a field with lots of potential with AI, so learning about this might be useful in understanding what the day-to-day of radiology will look like in the future.”	14 (32.6%)
Training on best practices of large language model (LLM) use (ie, prompt engineering, citation checking)	“How to interpret and use AI-based clinical decision support tools”	11 (25.5%)
Training on limitations, biases, and ethics of LLMs	“I think it would be helpful to address strategies to mitigate patient confidentiality/privacy concerns if being utilized in a patient chart”	4 (9.3%)
Exposure to domain-trained LLMs in medicine	“Focus should be on specific tools designed and trained in an academic framework for dedicated medical or academic tools”	5 (11.6%)
Interest groups	“I think that it might be useful to implement AI via coding clubs in medical school”	2 (4.7%)
Training by developers or experts in AI	“Lecture from an AI expert on the AI Tools available, their benefits and limitations, and their future use”	2 (4.7%)

## Discussion

Our survey collected data on how medical students used and perceived generative AI for medical education. Due to the low response rate, the findings of the survey likely represent the behaviors and perceptions of a subgroup of early technology adopter medical students in Ontario. Over half of the surveyed medical students used generative AI regularly (≥once weekly), with ChatGPT being the predominant model across all purposes, not limited to medical education. Generative AI is used primarily for medical knowledge acquisition, educational coursework, and personal communication. In the clinical setting, generative AI is utilized for medical knowledge acquisition and generating differential diagnoses. It is infrequently used to assist in documentation for healthcare provider and patient-facing purposes. Many respondents shared concerns over the accuracy and inherent biases of LLMs. Most students agreed that institutional access and formal education on generative AI are important factors for the safe use of generative AI. Nearly all the respondents demonstrated a willingness to use generative AI in their future medical practice.

The findings of this survey suggest increased enthusiasm and propensity for generative AI among a highly engaged medical student subgroup compared with the literature. A 2023 study by Tangadulrat reported that 76% of medical students had positive perceptions of the use of ChatGPT for clinical practice,^14^ while our study revealed that 92.8% of participants were willing to learn how to use generative AI and integrate it into their future practice. The buy-in suggests that this cohort of early-adopter medical students may perceive generative AI to have transformative utility and value for medical practice. This may imply that the incoming generation of physicians is increasingly trusting in and enthusiastic for the integration of novel technology in medicine. In 2023, Tangadulrat et al found that 62% of respondents have never used AI and that only 8% used it regularly,^14^ whereas a year later, we reported that only 21.1% of respondents did not use generative AI, and that 53.0% of respondents used it regularly. This suggests that over time, as LLMs become more refined, more students may be engaging with LLMs, and those who had engaged previously are perhaps using these tools more regularly. In clinical settings, many students reported using generative AI to review medical concepts (68.5%) and learn medical concepts (63.0%). It was used to a lesser extent to generate differential diagnoses (46.1%) and very rarely to assist in documentation (5.2%). This decreased use may reflect concerns of breaching patient confidentiality, as documentation and diagnostic support require inputs with more detailed patient information. Further, this may indicate that while early adopters are enthusiastic about generative AI, they remain hesitant to use it in contexts that more directly impact patient care. A 2024 study by Mizuta et al found ChatGPT-4 was able to generate a differential diagnosis when given complex case reports with 95.9% accuracy,^19^ alluding to its use as a supportive tool for generating differential diagnoses may be suitable. Interestingly, in future practice, 89.6% of participants reported a willingness to use generative AI to assist in documentation for healthcare worker-facing purposes. This discrepancy from current use may suggest medical students’ discomfort as trainees to employ AI to assist in medicolegal documentation, with greater anticipated comfort as an attending physician. This is a notable increase from the findings of Alkhaadi et al in 2023, where only 29.8% of medical students anticipated using ChatGPT to write patient notes,^12^ which perhaps again signifies an increasing comfort with the adoption of this technology. However, proofreading and human verification remain essential. Hayden et al reported that although ChatGPT can generate longer, higher quality notes than dictation and typing, 36% of documents contain erroneous information.^20^ Therefore, it is important to reinforce the necessity for human oversight by users, as they are ultimately responsible for any clinical decision and documentation put forward.^21^

Our survey also identified various barriers and concerns that may discourage the use of generative AI in medicine. Most respondents were concerned with the accuracy and reliability (91.6%) and the inherent biases (78.9%) of generative AI. These concerns align with the existing literature. A recent meta-analysis demonstrated ChatGPT had an integrated accuracy of 56% in addressing medical queries, which consisted of a combination of United States Medical Licensing Examination questions, clinical cases, and online examination banks.^22^ While generative AI has demonstrated immense promise as a medical resource, it is currently unable to fully capture the nuances and complexities of modern medicine. It should supplement and facilitate critical thinking rather than absolve it. This highlights the importance of educating medical students on safe practices of generative AI use, which was identified as one of the most common topics that respondents wish to formally learn in medical education regarding generative AI. Techniques such as “prompt engineering” to optimize LLM response, retrieving sources, and evaluating responses are integral methods to teach medical students to ensure appropriate, optimal use of generative AI for patient care.^23^

Currently, various studies have explored how LLMs may be integrated into the UGME curriculum to support test performance, clinical decision-making, and knowledge acquisition. A 2024 study evaluated the utility of OSCEai, a LLM designed to simulate clinical encounters.^24^ In this platform, users conduct a history via speech or text communication with the LLM and receive a feedback report based on the Calgary–Cambridge model for medical interviews. The study found that first-year medical students preferred OSCEai over traditional lecture-based methods, citing its value in providing on-demand feedback and supporting self-paced learning. A 2023 study by Meaney et al compared the performance of undergraduate medical students at the University of Toronto with that of ChatGPT-4 on a multiple-choice progress test designed to assess medical knowledge and clinical decision-making.^25^ ChatGPT-4 outperformed all years of medical students, achieving a score of 79%. The highest-performing cohort was third- and fourth-year students, with mean scores of 52.2% and 58.5%. A 2023 study compared the performance of ChatGPT-3.5 with that of first-year medical students on one of McMaster University's medical school examinations, which included clinical vignettes and short-answer questions. First-year students achieved a slightly higher average score (3.67 out of 5) compared to ChatGPT-3.5 (3.29 out of 5), although the difference was not statistically significant. Notably, several instructors grading the examinations frequently mistook ChatGPT-3.5 responses for those written by students. Large language models have demonstrated potential promise in supporting learners across Ontario in medical education.

All universities included in this study have implemented institution-wide policies to address the emergence of generative AI in education. Overall, these policies adopt a cautious stance, emphasizing that generative AI should not be used unless explicitly permitted by course instructors with specific guidance on acceptable versus prohibited applications.^26–31^ A consistent expectation across institutions is that students must both cite and describe the nature of generative AI use. To our current knowledge, no institution has released policies tailored specifically to undergraduate medical education. While our findings show that medical students are using generative AI in clinical settings for medical knowledge acquisition and to support differential diagnosis, the lack of clear policy guidance may make it difficult for learners to navigate these technologies safely and effectively. As outlined in Tables 3 and 5, many students expressed a desire for concrete examples of appropriate generative AI use, as well as training on how to apply these tools effectively in clinical settings. Notably, the postgraduate Family Medicine program at the University of Ottawa has permitted the use of AI scribes for second-year residents.^32^ This policy requires compliance with the College of Physicians and Surgeons of Ontario guidelines, which allow the use of AI scribes but place ultimate responsibility on the physician to ensure the accuracy and completeness of medical documentation, as well as to obtain patient consent.^33^

Our study is cross-sectional; however, generative AI models continually evolve with frequent releases of new features and capabilities, which can alter one's usage and perception of these tools. Future research should explore the temporal variation in the use and perceptions of generative AI throughout medical training, accounting for differences in clinical experience and the rapidly changing AI landscape. Our target population consisted of medical students, so the use and perspectives of residents and attending physicians should be explored in future studies. There is a notable difference in the reported use of generative AI in clinical settings compared with education, which might be attributed to the reduced accessibility and the risk of patient privacy breaches in the clinical setting. This remains a frontier, and the responsible adoption of generative AI in clinical settings may enhance elements of documentation and patient care. Our hope is that institutions across Ontario recognize the value and growing usage of generative AI in undergraduate medical education and develop robust policies and implement curricular adaptations to ensure optimal, responsible use of generative AI.

This study has several limitations. First, the response rate of 4.3% is low, and the findings of this survey are not necessarily generalizable to all medical students across Ontario. This may be partly attributable to institutional policies that restricted the modes of survey distribution to platforms infrequently accessed by medical learners. In some cases, survey distribution also coincided with the summer break, further contributing to the lower response rate. One of the institutions, Queen's University, only had one participant; as a result, this limits the applicability of this study's findings to that institution. Second, it should be noted that the participants in this study may represent a cohort of early technology adopters, thereby introducing a selection bias which might skew the data to show increased usage and enthusiasm for generative AI. Due to the rapidly evolving nature of generative AI, any cross-sectional findings quickly become outdated, reflecting only a snapshot of an ever-changing present. This may be particularly true with ChatGPT, as both the premium and free versions have frequent updates and new releases, thus its use may be particularly prone to dynamic changes. Finally, most participants were in their preclerkship years, during which there was limited exposure to clinical activities; therefore, the data may consequently underrepresent the usage of generative AI in clinical settings.

## Conclusion

Medical students reported the regular use of generative AI, primarily ChatGPT, to support their learning in medical education and to facilitate written communication. Medical students largely favor the adoption of generative AI in practice and wish to learn how to appropriately utilize these tools in practice. Moreover, many students shared concerns about the inherent biases in training data and the accuracy and reliability of outputs. This suggests that the use and integration of generative AI in the medical curriculum may enrich medical education and prepare the physicians of tomorrow with the skills to navigate these technological advancements in a responsible manner.

## Supplemental Material

sj-docx-1-mde-10.1177_23821205251391969 - Supplemental material for Perceptions and Use of Generative Artificial Intelligence in Medical Students: A Multicenter Survey Supplemental material, sj-docx-1-mde-10.1177_23821205251391969 for Perceptions and Use of Generative Artificial Intelligence in Medical Students: A Multicenter Survey by Cecilia Tran, Brett N. Hryciw, Sean William Moore, Alan Chaput and Andrew John Ervine Seely in Journal of Medical Education and Curricular Development

sj-docx-2-mde-10.1177_23821205251391969 - Supplemental material for Perceptions and Use of Generative Artificial Intelligence in Medical Students: A Multicenter Survey Supplemental material, sj-docx-2-mde-10.1177_23821205251391969 for Perceptions and Use of Generative Artificial Intelligence in Medical Students: A Multicenter Survey by Cecilia Tran, Brett N. Hryciw, Sean William Moore, Alan Chaput and Andrew John Ervine Seely in Journal of Medical Education and Curricular Development

sj-docx-3-mde-10.1177_23821205251391969 - Supplemental material for Perceptions and Use of Generative Artificial Intelligence in Medical Students: A Multicenter Survey Supplemental material, sj-docx-3-mde-10.1177_23821205251391969 for Perceptions and Use of Generative Artificial Intelligence in Medical Students: A Multicenter Survey by Cecilia Tran, Brett N. Hryciw, Sean William Moore, Alan Chaput and Andrew John Ervine Seely in Journal of Medical Education and Curricular Development

## References

[1] Jeyaraman M Ramasubramanian S Balaji S Jeyaraman N Nallakumarasamy A Sharma S . ChatGPT in action: harnessing artificial intelligence potential and addressing ethical challenges in medicine, education, and scientific research. World J Methodol. 2023;13(4):170-178. doi:10.5662/wjm.v13.i4.170 37771867 PMC10523250

[2] Cohen SA Brant A Fisher AC Pershing S Do D Pan C . Dr. Google vs. Dr. ChatGPT: exploring the use of artificial intelligence in ophthalmology by comparing the accuracy, safety, and readability of responses to frequently asked patient questions regarding cataracts and cataract surgery. Semin Ophthalmol. 2024;39(6):1-8. doi:10.1080/08820538.2024.2326058 38516983

[3] Hryciw BN Fortin Z Ghossein J Kyeremanteng K . Doctor-patient interactions in the age of AI: navigating innovation and expertise. Front Med. 2023;10. doi:10.3389/fmed.2023.1241508 PMC10498385 37711734

[4] Hryciw BN Seely AJE Kyeremanteng K . Guiding principles and proposed classification system for the responsible adoption of artificial intelligence in scientific writing in medicine. Front Artif Intell. 2023;6. doi:10.3389/frai.2023.1283353 PMC10687472 38035200

[5] Kung TH Cheatham M Medenilla A , et al. Performance of ChatGPT on USMLE: potential for AI-assisted medical education using large language models. PLOS Digit Health. 2023;2(2):e0000198. doi:10.1371/journal.pdig.0000198 PMC9931230 36812645

[6] Mir MM Mir GM Raina NT , et al. Application of artificial intelligence in medical education: current scenario and future perspectives. J Adv Med Educ Prof. 2023;11(3):133-140. doi:10.30476/JAMP.2023.98655.1803 37469385 PMC10352669

[7] Mohammad B Supti T Alzubaidi M , et al. The pros and cons of using ChatGPT in medical education: a scoping review. In: Healthcare Transformation With Informatics and Artificial Intelligence. IOS Press; 2023; 644-647. doi:10.3233/SHTI230580 37387114

[8] Xu X Chen Y Miao J . Opportunities, challenges, and future directions of large language models, including ChatGPT in medical education: a systematic scoping review. J Educ Eval Health Prof. 2024;21(6). doi:10.3352/jeehp.2024.21.6 PMC11035906 38486402

[9] Moldt JA Festl-Wietek T Madany Mamlouk A Nieselt K Fuhl W Herrmann-Werner A . Chatbots for future docs: exploring medical students’ attitudes and knowledge towards artificial intelligence and medical chatbots. Med Educ Online. 2023;28(1):2182659. doi:10.1080/10872981.2023.2182659 36855245 PMC9979998

[10] Stewart J Lu J Gahungu N , et al. Western Australian medical students’ attitudes towards artificial intelligence in healthcare. PLoS One. 2023;18(8):e0290642. doi:10.1371/journal.pone.0290642 PMC10470885 37651380

[11] Hosseini M Gao CA Liebovitz DM , et al. An exploratory survey about using ChatGPT in education, healthcare, and research. PLoS One. 2023;18(10):e0292216. doi:10.1371/journal.pone.0292216 PMC10553335 37796786

[12] Alkhaaldi SMI Kassab CH Dimassi Z , et al. Medical student experiences and perceptions of ChatGPT and artificial intelligence: cross-sectional study. JMIR Med Educ. 2023;9(1):e51302. doi:10.2196/51302 PMC10770787 38133911

[13] Pucchio A Rathagirishnan R Caton N , et al. Exploration of exposure to artificial intelligence in undergraduate medical education: a Canadian cross-sectional mixed-methods study. BMC Med Educ. 2022;22(1):815. doi:10.1186/s12909-022-03896-5 36443720 PMC9703803

[14] Tangadulrat P Sono S Tangtrakulwanich B . Using ChatGPT for clinical practice and medical education: cross-sectional survey of medical Students’ and Physicians’ perceptions. JMIR Med Educ. 2023;9(1):e50658. doi:10.2196/50658 PMC10770783 38133908

[15] Mehta N Harish V Bilimoria K , et al. Knowledge of and attitudes on artificial intelligence in healthcare: a provincial survey study of medical students. medRxiv. 2021. Preprint posted online: 2021.01.14.21249830. doi:10.1101/2021.01.14.21249830

[16] Tan S Xin X Wu D . ChatGPT in medicine: prospects and challenges: a review article. Int J Surg Lond Engl. 2024;110(6):3701-3706. doi:10.1097/JS9.0000000000001312 PMC11175750 38502861

[17] von Elm E Altman DG Egger M , et al. The Strengthening The Reporting Of Observational Studies in Epidemiology (STROBE) statement: guidelines for reporting observational studies. Ann Intern Med. 2007;147(8):573-577. doi:10.7326/0003-4819-147-8-200710160-00010 17938396

[18] Kiger ME Varpio L . Thematic analysis of qualitative data: AMEE guide No. 131. Med Teach. 2020;42(8):846-854. doi:10.1080/0142159X.2020.1755030 32356468

[19] Mizuta K Hirosawa T Harada Y Shimizu T . Can ChatGPT-4 evaluate whether a differential diagnosis list contains the correct diagnosis as accurately as a physician? Diagn Berl Ger. 2024;11(3):321-324. doi:10.1515/dx-2024-0027 38465399

[20] Baker HP Dwyer E Kalidoss S Hynes K Wolf J Strelzow JA . ChatGPT’s ability to assist with clinical documentation: a randomized controlled trial. J Am Acad Orthop Surg. 2024;32(3):123. doi:10.5435/JAAOS-D-23-00474 37976385

[21] Haltaufderheide J Ranisch R . The ethics of ChatGPT in medicine and healthcare: a systematic review on large language models (LLMs). NPJ Digit Med. 2024;7(1):1-11. doi:10.1038/s41746-024-01157-x 38977771 PMC11231310

[22] Wei Q Yao Z Cui Y Wei B Jin Z Xu X . Evaluation of ChatGPT-generated medical responses: a systematic review and meta-analysis. J Biomed Inform. 2024;151:104620. doi:10.1016/j.jbi.2024.104620 38462064

[23] Giray L . Prompt engineering with ChatGPT: a guide for academic writers. Ann Biomed Eng. 2023;51(12):2629-2633. doi:10.1007/s10439-023-03272-4 37284994

[24] Guo E Ramchandani R Park YJ Gupta M . OSCEai: personalized interactive learning for undergraduate medical education. Can Med Educ J. Published online 2025. doi:10.36834/cmej.79220 PMC12826823 41584941

[25] Meaney C Huang RS Lu K , et al. Comparing the performance of ChatGPT and GPT-4 versus a cohort of medical students on an official university of Toronto undergraduate medical education progress test. medRxiv. Preprint posted online September 14, 2023:2023.09.14.23295571. doi:10.1101/2023.09.14.23295571

[26] Guidelines on the use of Generative AI in Teaching and Learning. Academic excellence - office of the provost. Accessed September 13, 2025. https://provost.mcmaster.ca/generative-artificial-intelligence-2/task-force-on-generative-ai-in-teaching-and-learning/provisional-guidelines-on-the-use-of-generative-ai-in-teaching-and-learning/

[27] Generative artificial intelligence in the classroom: FAQ’s – Office of the vice-provost, innovations in undergraduate education. Accessed September 13, 2025. https://www.viceprovostundergrad.utoronto.ca/16072-2/teaching-initiatives/generative-artificial-intelligence/

[28] Teaching and learning statements, guidelines and resources | office of the provost and vice-principal. Accessed September 13, 2025. https://www.queensu.ca/provost/teaching-and-learning/statements-guidelines-and-resources/teaching-and-learning-statements

[29] Guidelines for the use of GenAI in teaching and learning | NOSM U. Accessed September 13, 2025. https://www.nosm.ca/about/administrative-offices/provost-and-vice-president-academic/artificial-intelligence-ai-guidelines/

[30] What is Western’s AI policy? Accessed September 13, 2025. https://www.uwo.ca/Guidance/Policy.html

[31] 2025. Academic integrity. Study. Accessed September 13, 2025. https://www.uottawa.ca/study/academic-integrity.

[32] 2025. Use of AI Scribes April 2025.pdf. Accessed September 13, 2025. https://www.uottawa.ca/faculty-medicine/sites/g/files/bhrskd401/files/2025-04/Use%20of%20AI%20Scribes%20April%202025.pdf.

[33] Using AI Scribes in your practice | dialogue - CPSO’s Publication for Ontario doctors. Accessed September 13, 2025. https://dialogue.cpso.on.ca/articles/using-ai-scribes-in-your-practice

