# Supplementary material for: Perceptions and Use of Generative Artificial Intelligence in Medical Students: A Multicenter Survey
Source: J Med Educ Curric Dev. 2025 Oct 29;12:23821205251391969. doi: 10.1177/23821205251391969 (PMC12576227; doi:10.1177/23821205251391969)
Supplement: sj-docx-1-mde-10.1177_23821205251391969 - Supplemental material for Perceptions and Use of Generative Artificial Intelligence in Medical Students: A Multicenter Survey [file sj-docx-1-mde-10.1177_23821205251391969.docx]

## **Supplemental Survey Instrument 1** Complete exploratory survey of how medical students use and perceive generative AI in medicine.

## Section 1: Implied Consent

**Implied Consent Form**

**Study Title:** **The Use and Perceptions of Artificial Intelligence (AI) in Undergraduate Education: A Multicenter Study**

**Principal Investigator:** Andrew J.E. Seely, Telephone: (613) 737-8899 ex. 74052

**OHSN-REB Number:** 20240208-01H

INTRODUCTION

You are being asked to participate because you are a medical student or resident with access to Generative Artificial Intelligence (AI) such as ChatGPT. This study examines how AI is utilized and perceived by medical students and residents in their education and training.

ARE THERE ANY CONFLICTS OF INTEREST?

There are no conflicts of interest to declare related to this study.

WHAT WILL HAPPEN DURING THIS STUDY?

Your participation in this study will require the completion of an anonymous survey. The survey asks questions about your experience with AI and your attitude towards its use in clinical care. This should take approximately 5 minutes of your time.

The information you provide is for research purposes only. Some of the questions are personal. You can choose not to answer questions if you wish.

Communication via e-mail is not absolutely secure. We do not recommend that you communicate sensitive personal information via e-mail.

VOLUNTARY PARTICIPATION AND WITHDRAWAL:

You do not have to be in this study if you do not want to be. You can choose to end your participation in this research (called withdrawal) at any time without having to provide a reason by closing your browser. However, once you submit the survey, it will not be possible to withdraw your information. The decision to complete this survey will not affect your academic performance or status as a medical student or resident at your institution.

RISKS AND/OR BENEFITS

Participation involves minimal risk to you. Some of the questions may however make you feel uncomfortable.

You may not receive direct benefit from participating in this study. We hope the information learned from this study will help adapt medical education in the future.

PRIVACY/CONFIDENTIATLITY:

The survey is anonymous which means that your answers will not be linked to you in any way.

Authorized representatives of the following organizations may look at your original research records at the site where these records are held, to check that the information collected for the study is correct and follows proper laws and guidelines.

- The Ottawa Health Science Network Research Ethics Board who oversees the ethical conduct of this study.
- Ottawa Hospital Research Institute to oversee the conduct of research at this location.

Information that is collected about you for the study (called study data) may also be sent to the organizations listed above. Your email, or other information that may directly identify you will not be used. The records received by these organizations may contain your gender, age, institution, specialty, race, and level of study.

This research study is collecting information on race and ethnicity as well as other characteristics of individuals because race is identified as a factor that can influence technology acceptance. Providing information on your race or ethnic origin is voluntary.

If the results of this study are published, your identity will remain confidential. It is expected that the information collected during this study will be used in analyses and published in journals.

Your anonymous data from this study may be used for other research purposes. If your study data is shared with other researchers, no information that links your study data directly to you will be available.

Even though the risk of identifying you from the study data is very small, it can never be completely eliminated.

COST AND/OR PAYMENT:

You will not be paid for being in this study, nor will there be any cost to you.

RIGHTS OF PARTICIPANTS

Your rights to privacy are legally protected by federal and provincial laws that require safeguards to ensure that your privacy is respected.

QUESTIONS:

If you have any questions about taking part in this study, you may contact Cecilia Tran ([cetran@ohri.ca](mailto:cetran@ohri.ca) ) or Dr. Andrew Seely ([aseely@toh.ca](mailto:aseely@toh.ca))

If you have questions about your rights as a participant or about ethical issues related to this study, you can talk to someone who is not involved in the study at all. Please contact The Ottawa Health Science Network Research Ethics Board, Chairperson at 613-798-5555 extension 16719.

CONSENT

By completing this survey your consent to participate is implied.

## Section 2: Utility of Generative AI

Generative AI is novel technology which can create novel content from existing datasets from the internet.

Text-to-Text models are chatting robots such as ChatGPT which use large learning models to generate natural dialogue (^1^).

Text-to-Image models are tools which synthesize realistic images and visual content based on user instruction (^2^).

**In this survey, we will use generative AI to refer to Text-to-Text and Text-to-Image tools.**

^1^ M., Madain, A., & Jararweh, Y. (2022). Chatgpt: Fundamentals, applications and social impacts. 2022 Ninth International Conference on Social Networks Analysis, Management and Security (SNAMS). https://doi.org/10.1109/snams58071.2022.10062688

^2^ Gozalo-Brizuela,R. & Garrido-Merchan, E. C. (2023). ChatGPT is not all you need. A State of the Art Review of large Generative AI models. arXiv. https://doi.org/10.48550/arXiv.2301.04655

1. Do you use generative AI (i.e. ChatGPT, Google Bard, Dall-E)?

- Yes
- No

*If the answer to question 1 is no, the question below will apply, then survey will skip to section 3:*

1. Select the reason you do not use generative AI.

- Never heard of it
- Never tried to use it
- Used it, but did not find it helpful
- Other:

*If the answer to question 1 is yes, all the following questions will apply:*

1. Select all the generative AI tools you use. [multi-select]

- ChatGPT (free version)
- ChatGPT (paid version)
- Bard
- Gemini
- Perplexity AI
- Glass AI
- Dall-E
- Dall-E-2
- Dall-E-3
- Runway
- Other:

1. Please indicate the percentage in which you use generative AI tools (I.e. ChatGPT: 80%, Glass AI: 20%).
2. How frequently do you use the selected generative AI from question 3 (non-academic related, academic related)?

- Less than 4 times a month
- Once a week
- 3-6 times a week
- Daily
- More than once daily

1. What language do you use for generative AI?

- English
- French
- Other:

1. Select all the activities you use generative AI for in education: [multi-select]

- Learn medical concepts (i.e. look up for the first time)
- Review medical concepts (i.e. confirm existing knowledge)
- Summarize scientific literature
- Summarize clinical guidelines
- Study resource for exams (i.e. creating notes, summaries, study guide)
- Exam preparation through self-testing (generate questions then receive feedback on your answer)
- Image generation to help with learning, testing, algorithm generation for approach to problems
- Other:

1. Select all the activities you use generative AI for in clinical settings: [multi-select]

- Learn medical concepts
- Review medical concepts
- Summarize scientific literature
- Summarize clinical guidelines
- Generating differential diagnoses
- Clinical decision making (i.e. assessment, management)
- Other:

1. Select all the activities you use generative AI for in communication in any capacity (compose, edit, paraphrase): [multi-select]

- Academic use (i.e. essays, applications, speeches, emails)
- Research purposes (i.e. emails, citations, literature reviews)
- Patient facing purposes (i.e. patient letters, patient information sheets)
- Doctor facing purposes (i.e. referrals, encounter notes, documentation in EMR)
- Personal use (i.e. emails, social media posts)
- Generating media (i.e. images, videos, posters, infographics, PowerPoints)
- Other:

*If participant selects generating media, the below question will apply:*

1. Please provide an example of the media you have used generative AI to synthesize (i.e. images, videos, posters, infographics, PowerPoints):
2. Do you pay for generative AI tools?

- Yes
- No

1. If no, what are the barriers that deter you? Select all that apply. [multi-select]

- Too expensive
- Little added value to paid versions
- Do not use it enough
- Other:

## Section 3: Perception and Attitude of Generative AI

1. Do you think generative AI should be implemented as resources or be taught in UGME and PGME curriculum?

- Yes
- No
- Other:

1. Are you willing to learn how to use generative AI and integrate it into your practice in the future, in any capacity?

- Yes
- No

1. If yes, select the tasks you would use generative AI tools to **assist** in? [multi-select]

- Written material for patient-facing purposes (i.e. patient letters, patient information sheets)
- Written material for health care provider-facing purposes (i.e. referrals, encounter notes)
- Generating differential diagnoses
- Approach to patient assessment
- Management and treatment
- Other:

1. What do you perceive as the enablers which would encourage your use of generative AI? [multi-select]

- Free or institutional access
- Integrated use within curriculum
- Training on how to use generative AI
- Use of an AI tool specific to medicine rather than an all-purpose tool
- Discussion groups on use
- Other

1. What do you perceive as the barriers which would discourage your use of generative AI?

- Unaware or unfamiliar of generative AI
- Associated negative stigma
- Cost of premium access
- Limitations of generative AI
- Concerns of accuracy and reliability
- Concerns of inherent biases within generative AI
- Other:

## Section 4: Participant Characteristics

1. What is your age?

- 18-24
- 25-34
- 35-44
- 45-54
- Other

1. What is your gender?

- Woman
- Man
- Non-binary

1. What is your race? [multi-select]

- Arab
- Black
- Chinese
- Filipino
- Japanese
- Korean
- Latin American
- South Asian (e.g., East Indian, Pakistani, Sri Lankan)
- Southeast Asian (e.g., Vietnamese, Cambodian, Laotian, Thai)
- West Asian (e.g., Iranian, Afghan)
- White
- Other group—specify

1. Indicate your year of study.

- 1^st^ year
- 2^nd^ year
- 3^rd^ year
- 4^th^ year
- 5^th^ year
- 6^th^ year

1. Indicate the institution you are enrolled in.

## Section 5: Open Forum

1. Do you have any **recommendations** as to how AI could be implemented in undergraduate medical education or residency training?
2. Please list and explain any **concerns** you have over the use of AI in medical training.
3. Are there **educational topics** regarding AI with regards to medicine you would like your institution to formally teach?
