# Supplementary material for: Perceptions and Use of Generative Artificial Intelligence in Medical Students: A Multicenter Survey
Source: J Med Educ Curric Dev. 2025 Oct 29;12:23821205251391969. doi: 10.1177/23821205251391969 (PMC12576227; doi:10.1177/23821205251391969)
Supplement: sj-docx-3-mde-10.1177_23821205251391969 - Supplemental material for Perceptions and Use of Generative Artificial Intelligence in Medical Students: A Multicenter Survey [file sj-docx-3-mde-10.1177_23821205251391969.docx]

**Figure 1** Types of large language models (multiple responses allowed) used by medical students (n=132)
